# Antipsychotic drugs and risks of myocardial infarction: a self-controlled case series study

**DOI:** 10.1093/eurheartj/ehu263

**Published:** 2014-07-08

**Authors:** Ruth Brauer, Liam Smeeth, Karim Anaya-Izquierdo, Adam Timmis, Spiros C. Denaxas, C. Paddy Farrington, Heather Whitaker, Harry Hemingway, Ian Douglas

**Affiliations:** 1 Department of Non-Communicable Disease Epidemiology, Faculty of Epidemiology and Population Health, London School of Hygiene and Tropical Medicine, Keppel Street, London WC1E 7HT, UK; 2 Department of Infectious Disease Epidemiology, Faculty of Epidemiology and Population Health, London School of Hygiene and Tropical Medicine, Keppel Street, London WC1E 7HT, UK; 3 National Institute for Health Research Biomedical Research Unit, Barts Health, London, UK; 4 Department of Epidemiology and Public Health, Clinical Epidemiology, University College London, 1–19 Torrington Place, London WC1E 7HB, UK; 5 Department of Statistics, The Open University, Milton Keynes MK7 6BJ, UK

**Keywords:** Myocardial infarction, Antipsychotic agents, Self-controlled case series, Case–control study

## Abstract

**Aim:**

Antipsychotics increase the risk of stroke. Their effect on myocardial infarction remains uncertain because people prescribed and not prescribed antipsychotic drugs differ in their underlying vascular risk making between-person comparisons difficult to interpret. The aim of our study was to investigate this association using the self-controlled case series design that eliminates between-person confounding effects.

**Methods and results:**

All the patients with a first recorded myocardial infarction and prescription for an antipsychotic identified in the Clinical Practice Research Datalink linked to the Myocardial Ischaemia National Audit Project were selected for the self-controlled case series. The incidence ratio of myocardial infarction during risk periods following the initiation of antipsychotic use relative to unexposed periods was estimated within individuals. A classical case–control study was undertaken for comparative purposes comparing antipsychotic exposure among cases and matched controls. We identified 1546 exposed cases for the self-controlled case series and found evidence of an association during the first 30 days after the first prescription of an antipsychotic, for first-generation agents [incidence rate ratio (IRR) 2.82, 95% confidence interval (CI) 2.0–3.99] and second-generation agents (IRR: 2.5, 95% CI: 1.18–5.32). Similar results were found for the case–control study for new users of first- (OR: 3.19, 95% CI: 1.9–5.37) and second-generation agents (OR: 2.55, 95% CI: 0.93–7.01) within 30 days of their myocardial infarction.

**Conclusion:**

We found an increased risk of myocardial infarction in the period following the initiation of antipsychotics that was not attributable to differences between people prescribed and not prescribed antipsychotics.

## Introduction

Antipsychotics are widely prescribed with a total of 9.3 million prescriptions dispensed in England in 2011.^1^ Their cardiovascular risk profile is of concern with users at an increased risk of death from cardiac causes and stroke.^2–6^ A recent study suggests that older patients with dementia are at an increased risk of myocardial infarction (MI) shortly after antipsychotic initiation,^7^ but the question of whether antipsychotics are associated with MI in the general population is unanswered.^8^ As the underlying risk of MI in patients with a major psychiatric illness is higher than in the general population, there is a great scope for bias when estimating treatment effects comparing people prescribed and not prescribed antipsychotic drugs.^9–11^ The aim of our study was to investigate this association using the self-controlled case series (SCCS) design, where comparisons are made within individuals who have both the event of interest and the exposure of interest, eliminating confounding due to between-person differences.^12^

## Methods

### Databases used

Data for this study were provided by the CArdiovascular disease research using Linked Bespoke studies and Electronic Records (CALIBER) programme, established in 2011 to provide evidence across different stages of research, in part by providing linkages of multiple electronic heath records.^13^ This study was based on linking the national myocardial infarction register [the Myocardial Ischaemia National Audit Project (MINAP)] to primary care records collected and archived by the UK Clinical Practice Research Datalink (CPRD).  Myocardial Ischaemia National Audit Project facilitates comparisons of MI management between hospitals in England and Wales in which patients with acute coronary symptoms are admitted since 1999.^13^ Validated MINAP data provide information on key clinical indicators with regard to myocardial ischaemia. Individual CPRD practices are linked with MINAP and all the patients from these practices are linked if they have an event recorded in MINAP. The CPRD contains the anonymized longitudinal electronic medical records of ∼8% of all UK patients.^14^ Almost 50% of all patients in the CPRD have at least 5 years of follow-up and data quality is high. This study used the medical records of patients registered at one of 264 MINAP-linked UK general practices (GPs), comprising 2 625 818 active patients in November 2010.

### Source population, outcome, and exposure

The source population was all the patients aged 18 years and older registered in the MINAP-linked CPRD. Patients had to have at least 1 year of enrolment at their GP before entry into the study. Follow-up was censored at the earliest of the date the patient left the practice, death date, switch of antipsychotic class, or the latest date of data collection. Patients with an MI were identified in the CPRD if they had a code of an incident MI in their medical file or if they were referred to the hospital with a code of incident MI between 1987 and March 2010. Patients with an MI were identified in MINAP, if they were discharged from the hospital with a diagnosis of an incident MI between January 2003 and August 2009. A validation study, using the same data sources, reported a high validity of CPRD MI diagnoses: the positive predictive value of MI as recorded in the CPRD database, using electrocardiographic and troponin recordings in MINAP as a gold standard, is 92%.^15^ Exposure was defined as a recorded prescription for an antipsychotic. For the purposes of this study, antipsychotics were classed as either first- or second-generation antipsychotics. First-generation agents included pimozide, butyrophenones, phenothiazines, and sulpiride. Second-generation antipsychotics included amisulpride, clozapine, olanzapine, quetiapine, and risperidone. Patients exposed to discontinued products or prochlorperazine only were excluded as the indications for prochlorperazine, including severe nausea, vomiting and vertigo, were not considered comparable with indications for other antipsychotics. We searched for records suggesting (i) schizophrenia and psychosis, (ii) mood disorders (including depression), (iii) dementia, and (iv) other psychiatric indications at the time of MI. The duration of each prescription was estimated by dividing the total quantity received by the numeric daily dose prescribed. The median length of 28 days was imputed if information on quantity or dose was missing. Where apparent treatment breaks occurred, a period of up to 60 days was allowed during which the patient was considered continuously exposed, allowing for medication stockpiling and non-adherence.

The study complies with the Declaration of Helsinki and was approved by the LSHTM Ethics committee (5743), CALIBER SOC and CPRD ISAC (10_073) and is registered with ClinicalTrials.gov (NCT01236274).

### Self-controlled case series study

**Figure 1:**
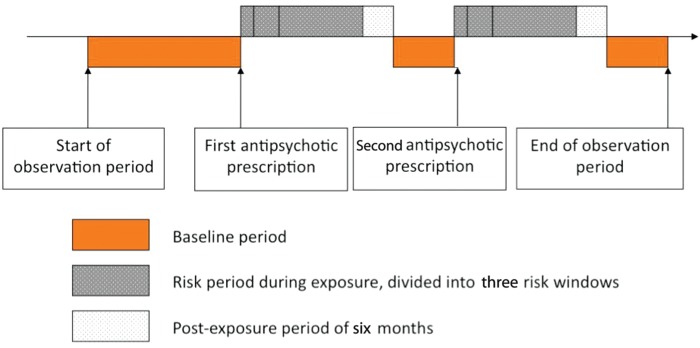
Self-controlled case series design ‘myocardial infarction and antipsychotics’.

The SCCS method is a type of a cohort study in which the relative risk is based on within-person comparisons rather than between-person comparisons, with each person contributing to both the exposed and unexposed observation time.^12^ This means the confounding effects of differences between patients are removed if these factors are fixed over the observation period. Time-varying confounders, such as age, can be controlled for. All the cases with a first recorded MI in CPRD or MINAP and a first recorded antipsychotic prescription 12 months after the start of the CPRD follow-up were selected as the study population. The observation time was divided into risk windows: (i) 0–30 days after starting treatment, (ii) 31–91 days after starting treatment, (iii) the remaining exposed time, followed by (iv) a post-exposure 6-month period divided into 60 day periods representing a gradual shift from full exposure to an entirely unexposed state; the baseline period comprised all remaining unexposed time (see *Figure 1*). Age was modelled explicitly in 2-year age bands from 40 to 90 years with the youngest (18–40 years) and oldest age group (>90 years) summarized. Subgroup analyses were performed for users of first- and second-generation antipsychotics, for patients with and without dementia and for patients with prior cardiovascular disease (CVD).

An important assumption underpinning the SCCS is that observation periods of individuals should end independently of the timing of the outcome. When this assumption is violated (e.g. the outcome increases the short-term risk of death), a recently developed extension of the method should be used. Correcting for non-random censoring of follow-up was necessary in this study as MI could lead to death and use of the standard SCCS method may result in bias.^16^

### Case–control study

Cases with a first recorded MI in the CPRD were randomly matched to up to five controls with no prior MI and actively registered with CPRD on the index date of the case by age, gender, and GP. Odds ratios (ORs) were calculated, comparing antipsychotic exposure in cases with controls. Exposure was defined as (i) any antipsychotic prescription within 90 days of the index date or (ii) having received a prescription >90 days before the event (former users) or (iii) first antipsychotic prescription within 30 days of the index date (new users). Subgroup analyses were performed for users of first-generation and second-generation antipsychotics. The risk estimates of the new users were compared with the results of the SCCS study.

Odds ratios were calculated both crude and adjusted for diagnoses of non-MI CVD, diabetes, hypertension, dyslipidaemia, CVD drugs use, smoking, alcohol use, and body mass index (BMI) at the time of the event. Clinical Practice Research Datalink medical records were used to determine covariates using coding algorithms developed by the CALIBER programme.

### Secondary analyses

Several additional analyses were performed: (i) All SCCS analyses were performed using both the standard and extended methods, to assess the impact of follow-up time censoring. (ii) Since MINAP outcomes were not included in the case–control study, the SCCS was repeated excluding MINAP outcomes for comparability. (iii) The 60-day period bridging the gap between prescriptions was shortened to 30 days or completely omitted. (iv) A separate SCCS analysis was conducted to report on the association between the use of prochloperazine and the risk of MI. (iv) The effect of excluding patients with missing data was investigated.

### Statistical methods

Incidence rate ratios for the SCCS study were calculated using conditional Poisson regression. For the case–control study conditional logistic regression was used to compute the crude and adjusted OR estimates. Never use of antipsychotics was used as a baseline comparator. Analyses were conducted using Stata, v11 (StataCorp, College Station, TX, USA). The modified case series design included the same parameters as the standard SCCS method: a parameter for the exposure of interest and a parameter for age, with the age parameter containing a component describing the short-term impact of the event of interest on censoring. A mixed exponential and Weibull model was used to model the density of age at the end of observation, which depended on the age at the occurrence of the event and individual characteristics. All modified case series analyses were performed in R, version 2.13.2.

## Results

### Identification of myocardial infarction cases

In the MINAP-linked CPRD database, 39 345 patients had an incident MI. Patients with <12 months of follow-up and patients who could not be matched to a suitable control were excluded (*n* = 4147); 35 198 cases were successfully matched to 137 919 controls.

**Figure 2:**
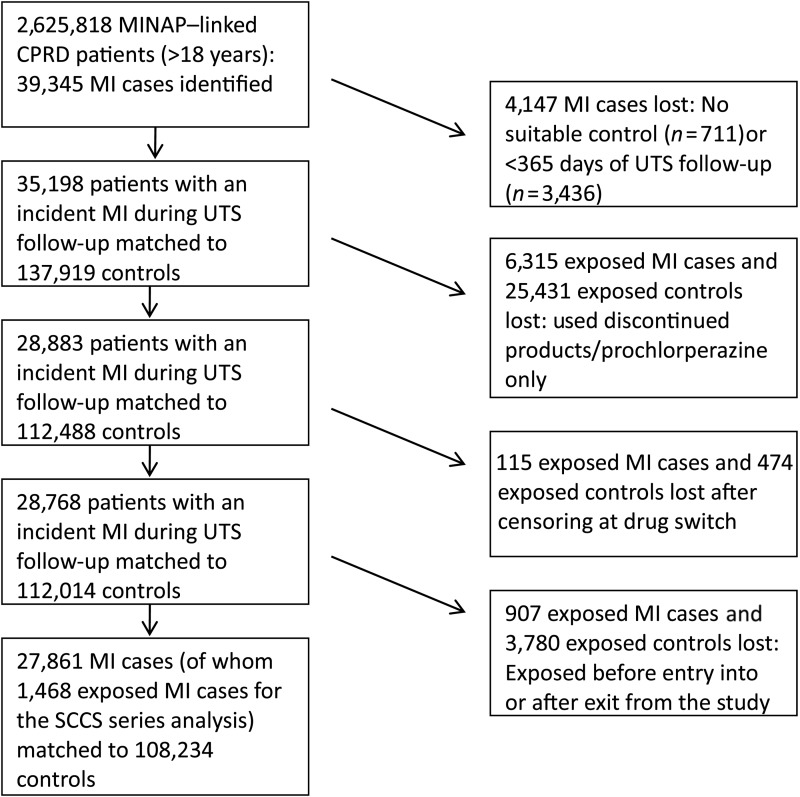
Flow diagram showing patient population for the case–control study ‘myocardial infarction and antipsychotics’.

Within this primary study population, 31 746 patients were exposed to prochlorperazine and/or discontinued products and were excluded, as were 115 cases with an incident MI after their censoring date (switch of antipsychotic drug). Lastly, 4687 patients (907 MI cases) were excluded as they were prevalent users. A flow diagram showing how patients were selected is shown in *Figure 2*.

### Results self-controlled case series

Ten thousand and five-hundred and seventy patients received an antipsychotic and had an MI recorded in either CPRD or MINAP. Twenty-four patients used first- and second-generation agents simultaneously and were excluded, leaving 1546 patients (*Table 1*). The median observation period was 11 years with a single exposure period lasting, on average, 3 months.

**Table 1:** Demographic details and distribution of cardiovascular and behavioural risk factors of the self-controlled case series study population at the time of the recording of an incident myocardial infarction

	Cases with a first recorded MI and antipsychotic prescription
Number	1546
Age (years) (SD)	70.24 (13.03)
Gender (%)	
Males	56.02
Females	43.98
Cardiovascular comorbidities (%)	
Atherosclerotic disease (including stroke, peripheral arterial disease)	805 (52.0)
The use of antiplatelets/anti-coagulants (excluding aspirin)	195 (12.6)
Aspirin	608 (39.2)
Hypertension (including the use of antihypertensive drugs)	1056 (68.3)
Diabetes mellitus (including the use of diabetic drugs)	231 (14.9)
Dyslipidaemia (including the use of lipid-lowering drugs)	376 (24.3)
Psychiatric co-morbidities (%)	
Schizophrenia and psychotic episodes	89 (5.8)
Mood disorders (including depression)	557 (36)
Other^a^	79 (5.1)
Dementia	117 (7.6)
Behavioural risk (%)	
Smoking (%)	
Current	423 (27.4)
Ex	541 (35.0)
Non	526 (34.0)
Missing	56 (3.6)
Alcohol (%)	
Excessive drinking	128 (8.3)
Current	913 (59.1)
Ex	66 (4.3)
Non	240 (15.5)
Missing	199 (12.9)
BMI (%)	
BMI >25	762 (49.3)
BMI <25	528 (34.2)
Missing	256 (16.6)
Antipsychotic prescription (%)	
First generation	670 (43.3)
Second generation	86 (5.6)

Figures are numbers of patients and percentages.

^a^Other psychiatric co-morbidities include obsessive-compulsive disorder, phobias, and recorded visits to the psychiatrist without a recorded indication.

Using the extended SCCS design, there was evidence of an increased risk of MI during the first 30 days after the first recorded prescription of a first- generation antipsychotic [incidence rate ratio (IRR) 2.82, 95% confidence interval (CI) 2.0–3.99] and during the first 30 days following a repeat prescription (IRR: 1.95, 95% CI: 1.19–3.21) (*Table 2*). There was similar evidence of an association between the prescription of second-generation agents and an increase in the risk of MI during the first month of use (IRR: 2.5, 95% CI: 1.18–5.32) but not thereafter (see *Table 2*). There was some evidence of an increase in risk of an incident recorded MI during the second and third month of exposure to first-generation antipsychotics (IRR: 1.41, 95% CI: 1.04–1.9), and some evidence of an increase in risk during any remaining exposed time thereafter for both first- (IRR: 1.47, 95% CI: 1.12–1.93) and second-generation (IRR: 1.75, 95% CI 1.06–2.87) antipsychotics. The relative risk of MI decreased during the 6-month post-exposure period after the use of second- generation agents (*Table 2*).

**Table 2:** Results self-controlled case series

Type of anti-psychotic	Exposure	Patient years	n MIs	Crude rate-ratio for MI [95% confidence interval (CI)]	Age-adjusted rate ratio for MI (95% CI) corrected for censoring
First generation	Unexposed	11 748	1021	Baseline	Baseline
	Exposed periods first 1–30 days of exposure	94	35	2.85 (2.02–4.02)	2.82 (2.0–3.99)
	Exposed periods 1–30 days for subsequent episodes of exposure^a^	97	17	2.04 (1.25–3.34)	1.95 (1.19–3.21)
	Exposed periods 31–90 days	330	49	1.44 (1.07–1.94)	1.41 (1.04–1.9)
	Exposed periods >90 days	789	104	1.57 (1.21–2.06)	1.47 (1.12–1.93)
	Post-exposure period 1–60 days	282	31	1.17 (0.81–1.68)	1.15 (0.8–1.66)
	Post-exposure period 61–120 days	239	34	1.53 (1.08–2.17)	1.52 (1.07–2.16)
	Post-exposure period 121–180 days	215	15	0.76 (0.46–1.28)	0.76 (0.45–1.27)
Second generation	Unexposed	1927	175	Baseline	Baseline
	Exposed periods first 1–30 days of exposure	19	8	2.75 (1.31–5.76)	2.5 (1.18–5.32)
	Exposed periods 1–30 days for subsequent episodes of exposure^a^	5	0	na	na
	Exposed periods 31–90 days	42	7	1.22 (0.56–2.7)	1.1 (0.49–2.45)
	Exposed periods >90 days	208	40	2.06 (1.32–3.21)	1.75 (1.06–2.87)
	Post-exposure period 1–60 days	22	5	1.93 (0.77–4.85)	1.74 (0.67–4.46)
	Post-exposure period 61–120 days	18	3	1.41 (0.44–4.55)	1.3 (0.4–4.25)
	Post-exposure period 121–180 days	15	2	1.19 (0.29–4.88)	1.13 (0.27–4.7)

^a^Exposure after treatment gaps.

There was no evidence that the risk differed between-patients diagnosed with dementia and those without: The IRR was 3.1 (95% CI: 1.0–9.6) during the first month of use of first-generation agents for patients with dementia (*n* = 83) and 2.73 (95% CI: 1.9–3.93) for patients without dementia (*n* = 1223). There were insufficient data to estimate the risk of MI in users of second-generation agents diagnosed with dementia (*n* = 34).

For users of first-generation antipsychotics, there was no evidence of any difference in risk between patients with recorded CVD prior to their MI (IRR: 2.7, 95% CI: 1.69–4.31, *n* = 681) and those without (IRR: 2.72, 95% CI: 1.62–4.59, *n* = 625). Some differences were found between users of second-generation antipsychotic agents with (IRR: 2.94, 95% CI: 1.13–7.64, *n* = 124) and without prior CVDs (IRR: 1.82, 95% CI: 0.52–6.29, *n* = 116).

### Comparison results self-controlled case series and case–control study

About 27 861 cases with an incident MI in the CPRD were matched to 108 234 controls (*Table 3*). Around 2% of patients were exposed to antipsychotics during their follow-up period. Most (*n* = 2521) were exposed to first-generation antipsychotics and phenothiazines were the most prescribed with 1552 exposed. Psychiatric morbidities, such as schizophrenia and major depressive episodes, were more often recorded among cases than controls. Cases were more often diagnosed with atherosclerotic disease, hypertension, diabetes, dyslipidaemia, and overweight at the time of their MI as well as more likely to smoke than the control population.

**Table 3:** Demographic details and distribution of cardiovascular and behavioural risk factors of study population at the time of the recording of an incident myocardial infarction

	Cases	Controls	*P*-value
Number	27 861	108 234	
Age (years) (SD)	68.69 (13.1)	67.14 (13.0)	<0.001
Gender (%)			
Males	65.97	68.8	<0.001
Females	34.03	31.2	
Cardiovascular comorbidities^a^ (%)			
Atherosclerotic disease (including stroke, peripheral arterial disease)	11 664 (41.9)	20 933 (19.3)	<0.001
The use of antiplatelets/anti-coagulants	2248 (8.1)	5158 (4.8)	<0.001
Hypertension (including the use of antihypertensive drugs)	16 475 (59.1)	43 341 (40.0)	<0.001
Diabetes mellitus (including the use of diabetic drugs)	3781 (13.6)	7628 (7.1)	<0.001
Dyslipidaemia (including the use of lipid-lowering drugs)	5925 (21.3)	14 546 (13.4)	<0.001
Psychiatric co-morbidities^a^ (%)			
Schizophrenia and psychotic episodes	259 (0.9)	747 (0.7)	<0.001
Mood disorders (including depression)	6708 (24.1)	21 288 (19.7)	<0.001
Other^b^	748 (2.7)	2322 (2.1)	<0.001
Dementia	388 (1.4)	1445 (1.3)	0.028
Behavioural risk (%)			
Smoking (%)			
Current	7456 (26.8)	20 053 (18.5)	<0.001
Ex	9405 (33.8)	33 645 (31.1)	
Missing	1970 (7.1)	9915 (9.2)	
Alcohol—excessive drinking (%)			
Current	2314 (8.3)	10 009 (9.2)	<0.001
Ex	897 (3.2)	2329 (2.2)	
Missing	3954 (14.2)	19 393 (17.9)	
BMI (%)			
>25	14 992 (53.8)	52 083 (48.1)	<0.001
Missing	4529 (16.3)	21 834 (20.2)	
Antipsychotic prescription (%)			
First generation	660 (2.4)	1861 (1.7)	<0.001
Second generation	64(0.2)	200 (0.2)	

Figures are numbers of patients and percentages.

^a^All co-morbidities in the conditional logistic regression were considered as dichotomous.

^b^Other psychiatric co-morbidities include obsessive-compulsive disorder, phobias and recorded visits to the psychiatrist without a recorded indication.

The results of the case–control study are shown in *Table 4*. There was evidence of an increased risk of MI up to 90 days after the receipt of a prescription for a first-generation antipsychotic agent [OR: 1.38, 95% confidence interval (CI) 1.16–1.64]. There was no evidence of an association between the current use of second-generation agents and the recording of an MI (OR: 1.07, 95% CI: 0.73–1.57). There was no evidence of an association between the former use of any antipsychotic agent (use of antipsychotics up 90 days before the event date) and the recording of an MI (OR: 0.91, 95% CI: 0.81–1.02 for first-generation agents and OR: 1.16, 95% CI: 0.71–1.9 for second-generation agents).

**Table 4:** Results case–control study

Exposure^a^	*n* controls	*n* MIs	Crude odds ratio for MI	Adjusted^b^ odds ratio for MI (95% CI)
First-generation agents				
Unexposed	105 802	27 113	Baseline	Baseline
Exposed 1–90 days before MI	487	214	1.56 (1.32–1.84)	1.38 (1.16–1.64)
Exposed > 90 days before MI	1372	437	1.14 (1.02–1.27)	0.91 (0.81–1.03)
First exposure 1–30 days before MI	35	32	3.2 (1.96–5.22)	3.19 (1.9–5.37)
Second-generation agents				
Unexposed	103 785	27 069	Baseline	Baseline
Exposed 1–90 days before MI	127	39	1.13 (0.78–1.62)	1.07 (0.73–1.57)
Exposed >90 days before MI	71	25	1.32 (0.83–2.1)	1.16 (0.71–1.9)
First exposure 1–30 days before MI	12	7	2.18 (0.85–5.6)	2.55 (0.93–7.01)

^a^Exposed 1–90 days before MI: Having received a prescription within −0 to 91 days before the event/exposed >90 days before MI: having received a prescription >91 days before the event.

^b^Adjusted for diagnoses of non-MI CVD, diabetes, hypertension, dyslipidaemia, CVD drugs use, smoking, excessive alcohol use, and overweight.

The association between the risk of MI and antipsychotic exposure during the first 30 days in the SCCS study was compared with the risk of MI and exposure during a similar risk window of the case–control study (first prescription received 1–30 days before a recorded MI). For first-generation agents, the results of the case–control study (OR: 3.19, 95% CI: 1.9–5.37) were very similar to the results of the modified SCCS study (IRR: 2.82, 95% CI: 2.0–3.99). For second-generation agents, the case–control study results (OR: 2.55, 95% CI: 0.93–7.01) were also very similar to those of the SCCS (IRR: 2.5, 95% CI: 1.18–5.32).

### Results secondary analyses

Qualitatively, the secondary SCCS analyses gave similar results to those obtained in the primary analysis (see Supplementary material online). The results of the secondary case–control analysis, using complete data only, showed strong evidence of an increased risk of MI up to 90 days after the receipt of a prescription for a first-generation antipsychotic agent (OR: 1.52, 95% CI: 1.21–1.92), but no evidence of an increase after receipt of a second- generation prescription (0.74, 95% CI: 0.46–1.21).

## Discussion

We investigated the relationship between the use of antipsychotics and the risk of an MI by performing a SCCS study and also a classical case–control study for comparative purposes. Regardless of the study design used, we found evidence that new users of antipsychotics were at an increased risk of MI during the first month of drug use. The increased risks were similar among those with and without diagnosed dementia or prior CVD.

### Comparison with previous studies

The increased risk of cardiac arrhythmias caused by antipsychotic drug use has been shown in previous studies.^6,17,18^ While the mechanism for the increase in risk of MI is not known, our results suggest that patients with a susceptibility may experience an MI early on in the treatment, independent of a history of cardiac disease, which could point to a triggering effect caused by drug-induced changes in heart rate.

Few studies have looked at the association between antipsychotics and MI. However, our results are similar to those of Pariente *et al*.^7^ who found that older patients using cholinesterase inhibitors were at an increased risk of MI when concomitant treatment with antipsychotics was initiated. They also found a strong increase in the risk of MI during the first month of antipsychotic use [RR: 1.78 (1.26–2.52)] after which the risk was similar to unexposed periods. Furthermore, our results are in agreement with the results of a cohort study conducted by Enger *et al*. who reported a five-fold higher risk of MI in users of first-generation antipsychotic agents compared with non-using control subjects.^19^ Differences in the strength of the effect may be explained by the inclusion of both prevalent and incident cases of MI and users of antipsychotics diagnosed with schizophrenia only by Enger *et al*.

In contrast, Nakagawa *et al*. reported no increase in risk in the first time antipsychotic users up to 90 days before a first recorded hospitalization for MI [adjusted relative risk: 0.98 (0.88–1.09) for second-generation agents and 0.99 (0.96–1.03) for first-generation agents].^20^ We believe that these differences in results may partly be explained by the exclusion of non-hospitalized MI cases by Nakagawa *et al*. A recent Canadian study showed that while patients with schizophrenia visit their general practitioner more often than patients without schizophrenia, they receive less specialized cardiac care.^21^ By limiting the outcome to events recorded in the hospital, we believe that some myocardial events may have been overlooked.

While we did not compare the increase in risk of MI between users of first- and second-generation antipsychotic agents, strong evidence of an increase in risk after receipt of a first-generation antipsychotic was consistently found regardless of the study design used. The point estimates for first-generation agents were higher than those reported for second-generation agents. The point estimate reported in a study by Huybrechts *et al*., in which antipsychotic-using US nursing home residents were followed up for 180 days, suggests that the initiation of first-generation antipsychotic use is associated with an increased risk of MI compared with the use of second- generation agents (propensity score-adjusted hazard ratio: 1.23, 95% CI: 0.82–1.82).^22^

### Strengths and limitations

Our study population was relatively large, representative of the UK population and included patients with and without dementia. We included incident antipsychotic users only and censored follow-up if patients switched between first- and second-generation antipsychotics. We were therefore able to analyse the effects of both types of antipsychotic separately. While we could not identify the precise indication for antipsychotics, we found that 87% of patients had a recorded psychiatric indication for antipsychotics with 75% of these recorded within a year of the first prescription. We tried to avoid extreme variability in underlying diagnoses by excluding patients prescribed prochlorperazine only. Results of secondary analyses showed a similar increase in risk of MI for users of prochlorperazine only, which suggests that antipsychotic agents may be associated with a temporary increase in risk of MI irrespective of the underlying diagnosis.

We used a recently developed extended version of the SCCS design as with the standard design bias may sometimes be introduced when the outcome of interest increases the short-term risk of death.^23^ Although MI clearly increases the risk of death, in this instance our comparison of the standard and designs gave very similar results, suggesting that such bias was avoided.

As with all studies using GP prescription data, we were not able to confirm that medications were obtained or used as directed and non-adherence may particularly have affected the results of the SCCS as accurate recording of the timing of exposure and outcomes is an important underlying assumption of case-only designs. A surprising increase in the risk of MI was found during the 60–120 days after the end of the last recorded prescription of a first-generation antipsychotic agent. This may suggest that the date we assigned as the end of a treatment course was underestimated due to medication stockpiling and non-adherence in some instances. Alternatively, multiple testing may have led to chance findings.

Two study designs were used with different strengths and weaknesses. The main advantage of the SCCS over other observational study designs is that patients act as their own control. As the results of the case–control study were similar to the results of the SCCS, confounding by known variables was unlikely to have had a distinct effect on our study results. We looked for evidence that antipsychotic initiation may have coincided with other risk factors for MI that change with time as an alternative explanation for our results: In the week of the first antipsychotic prescription, we identified 11 patients also receiving a first ever prescription for another medication and four other patients were newly diagnosed with diseases such as urinary infections. Excluding these 15 patients from the main analyses had no material effect on the results. This suggests other possible causes of MI did not tend to correlate with antipsychotic initiation. We acknowledge that protopathic bias cannot be excluded, by which some patients may have received a prescription for an antipsychotic due to prodromal symptoms of an MI which was then diagnosed after antipsychotic treatment started. While the possibility of drug interactions or a temporal change in underlying health state at the time of prescription cannot be ruled out, the greatest association between antipsychotics and MI was seen with the first ever exposure. Later exposures would also be expected to occur during similar changes in underlying health or with similar multiple prescriptions, suggesting a hypothesized drug effect occurring early on during exposure.

### Conclusions

The risk of MI is around two- to three-fold higher in the month following the initiation of antipsychotic treatment. The increased risk was not attributable to differences between people prescribed and not prescribed the drugs. Based on the results of the current study, the size of the observed risk could be similar to that seen for COX2 inhibitors.^24^ As antipsychotics are an effective intervention for some major psychiatric conditions, the relatively small increased absolute risk of MI is unlikely to alter their benefit–risk balance when used appropriately.^25^ However, when used in unlicensed indications without proven efficacy, such as dementia, the balance of risks and benefits is likely to be less favourable.

## Supplementary material

Supplementary material is available at *European Heart Journal* online.

## Funding

R.B. was funded by Amgen Limited in the context of the Pharmacoepidemiological Research on Outcomes of Therapeutics by a European Consortium (PROTECT) project. I.D. is funded by a Medical Research Council Methodology Fellowship, L.S. is funded by a Wellcome Trust Fellowship and C.P.F. is supported by a Royal Society/Wolfson research merit award. CALIBER is funded by a Wellcome Trust project grant (086091/Z/08/Z), a National Institute of Health Research (NIHR) programme grant (RP-PG-0407-10314), and a consortium of 10 UK government and charity funders (MR/K006584/1), led by the Medical Research Council (MRC), which funds the Centre for Health service and Academic Partnership in Translational Electronic health records Research (CHAPTER). The funders are Cancer Research UK (CRUK), Chief Scientist Office, Scottish Government Health Directorates (CSO), Engineering and Physical Sciences Research Council (EPSRC), Economic and Social Research Council (ESRC), National Institute for Health Research (NIHR), National Institute for Social Care and Health Research (NISCHR) and The Wellcome Trust. The views and opinions expressed therein are those of the authors and do not necessarily reflect those of the NIHR PHR Programme or the Department of Health. Funding to pay the Open Access publication charges for this article was provided by the Wellcome Trust.

**Conflict of interest:** none declared.
